# Supplementary figures and images for: Metabolite Variation between Nematode and Bacterial Seed Galls in Comparison to Healthy Seeds of Ryegrass Using Direct Immersion Solid-Phase Microextraction (DI-SPME) Coupled with GC-MS
Source: Molecules. 2023 Jan 13;28(2):828. doi: 10.3390/molecules28020828 (PMC9864257; doi:10.3390/molecules28020828)

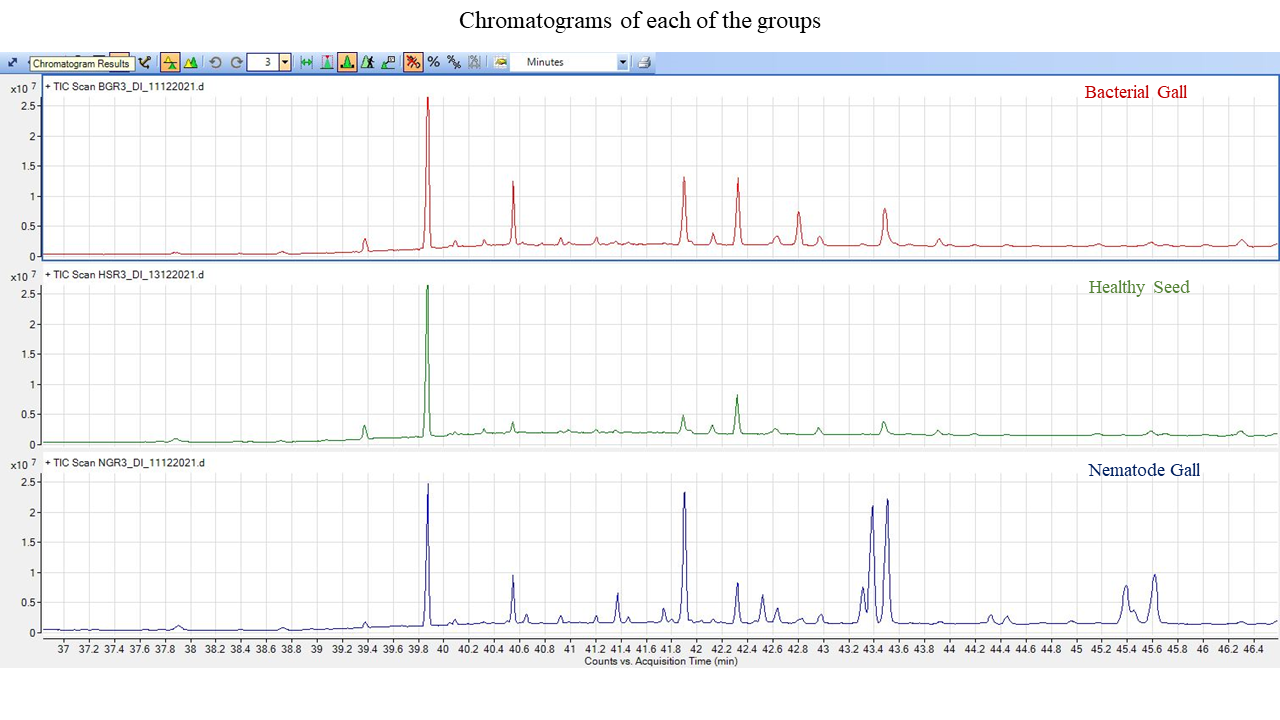

Supplement: Supplementary file 1 [file molecules-28-00828-s001.zip › Figure S1.png]

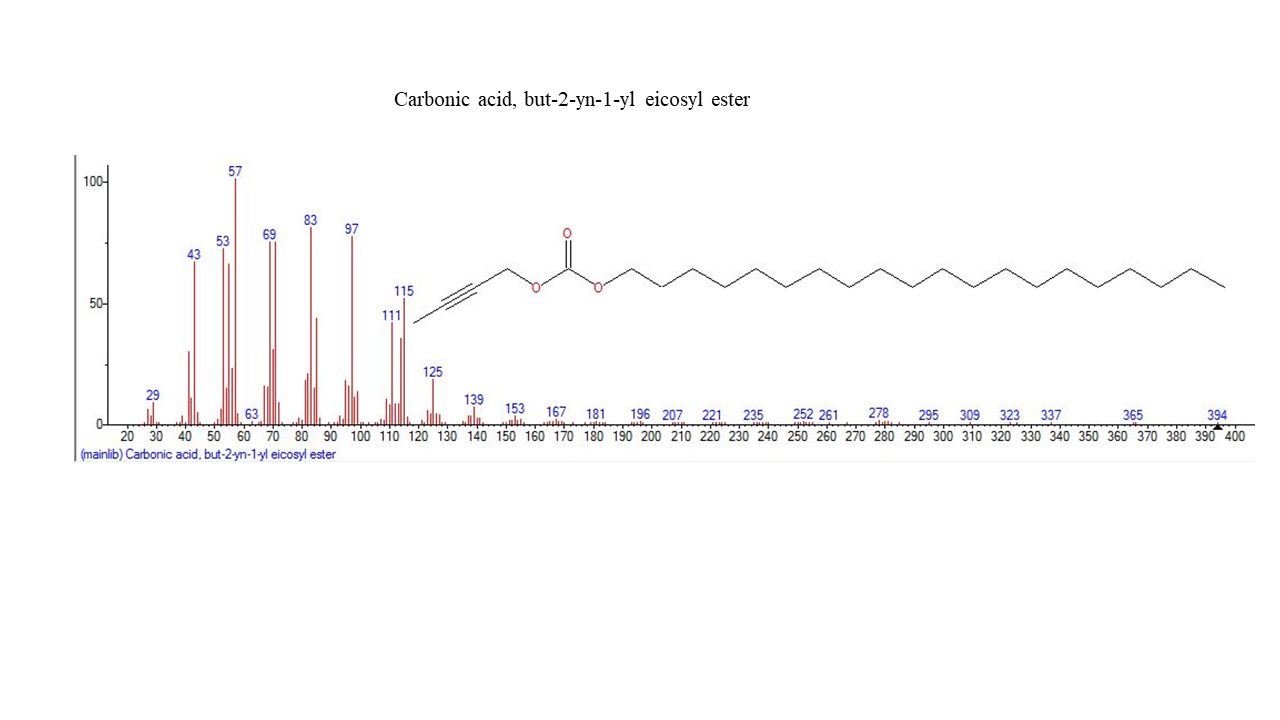

Supplement: Supplementary file 1 [file molecules-28-00828-s001.zip › Figure S2.png]

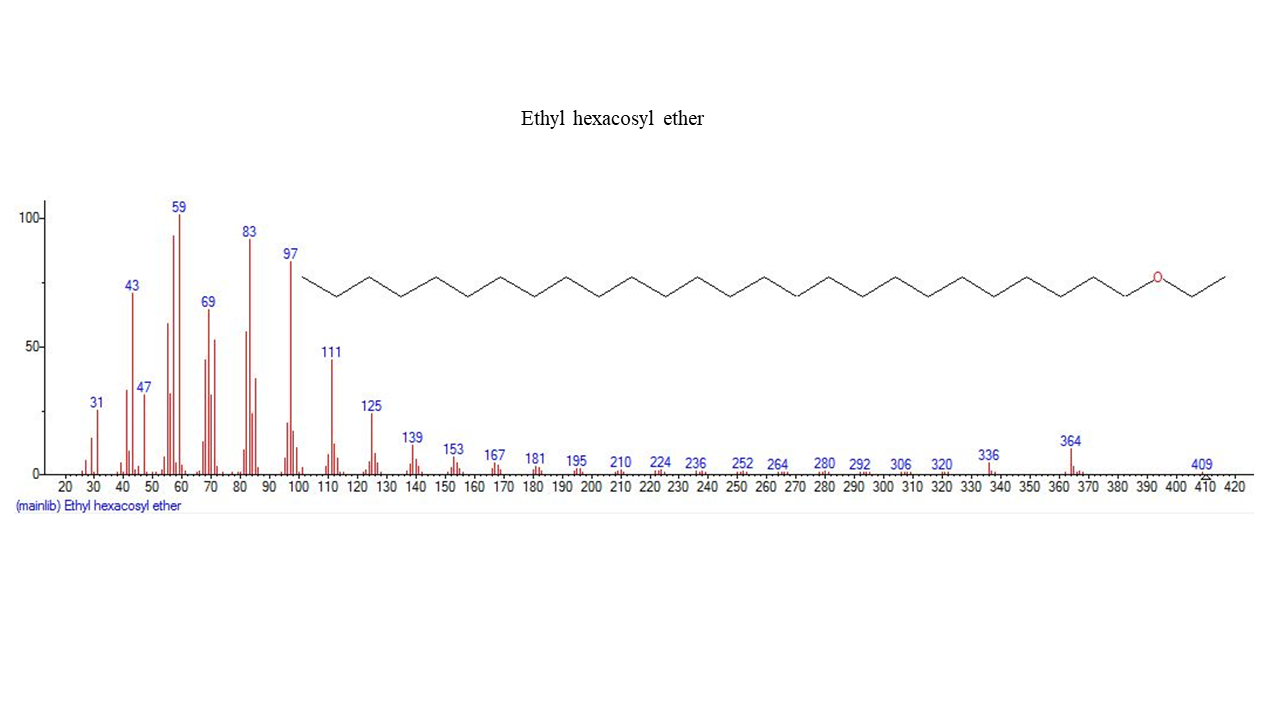

Supplement: Supplementary file 1 [file molecules-28-00828-s001.zip › Figure S3.png]
